# Öffentliche Mobilität gestalten – Die Mobilitätsberichterstattung

**DOI:** 10.1007/978-3-658-32106-2_11

**Published:** 2020-10-24

**Authors:** Sven Hausigke, Carolin Kruse

**Affiliations:** TU Berlin, Berlin, Germany; Technische Universität Berlin, Berlin, Deutschland

## Abstract

Wie mobil eine Person ist, wird bestimmt von der Anzahl und Qualität ihrer Möglichkeiten – dem Möglichkeitsraum. Die Mobilität von Menschen ist daher von den äußeren, öffentlichen Einflussfaktoren abhängig, die sich aus dem Verkehrssystem selbst als auch aus den räumlichen, gesellschaftlichen und ökonomischen Einflussfaktoren ergeben. Um Mobilität für alle zu gestalten, sind somit die Bedarfe der Bevölkerung und ihre bestehenden Möglichkeitsräume in der Verkehrsplanung zu berücksichtigen.

## Einleitung

Wie mobil eine Person ist, wird bestimmt von der Anzahl und Qualität ihrer Möglichkeiten – dem Möglichkeitsraum. Die Mobilität von Menschen ist daher von den äußeren, öffentlichen Einflussfaktoren abhängig, die sich aus dem Verkehrssystem selbst als auch den räumlichen, gesellschaftlichen und ökonomischen Einflussfaktoren ergeben (Schwedes et al. 2018, S. 5). Um Mobilität für alle zu gestalten, sind somit die Bedarfe der Bevölkerung und ihre bestehenden Möglichkeitsräume in der Verkehrsplanung zu berücksichtigen.

Auch wenn es zunächst so scheint, dass mit dem sukzessiven Ausbau des Straßennetzes – „bis ins kleinste Dorf“ (Gall 2005) – der Mobilitätsbedarf der Menschen gedeckt wird, ist festzustellen, dass hierdurch auch ein limitiertes Angebot geschaffen wurde: Eine monomodale Abhängigkeit vom Straßennetz und dem Auto. In den Nachkriegsjahren wurden in Städten der Bundesrepublik durch eine autoorientierte Verkehrsplanung Straßen für das individuelle Fahrzeug geschaffen und gleichzeitig der öffentliche Verkehr vernachlässigt oder gar abgebaut. Dies ist am Beispiel von Berlin gut zu erkennen. In Westberlin wurde die bestehende Straßenbahninfrastruktur demontiert und dafür Straßen und Autobahnen errichtet; in Ostberlin hingegen wurde die bestehende Straßenbahninfrastruktur weiter betrieben. Die negativen Folgen hieraus sind bekannt: schlechte Luft, Treibhausgase, Ressourcenverbrauch, Stress und Übergewicht, um nur einige zu nennen. Die Straßenbahn fand erst in der Verkehrspolitik der Nachwendezeit Einzug in das Westberliner Stadtbild.

Mit dem ansteigenden Motorisierungsgrad (BMVI 2019; UBA 2020) hat die Verkehrsplanung vor allem die Bedarfe der ‚neuen‘ Autofahrenden gedeckt, ohne zu hinterfragen, ob die Menschen tatsächlich das Auto bevorzugen, weil sie es möchten oder weil sie nicht mehr anders können. Nach nun über 80 Jahren der Förderung des Kfz und der noch immer häufig vorgenommenen Anpassungsplanung im Verkehr ist es überfällig, alle Bedarfe der Bevölkerung in der Verkehrsplanung endlich in den Mittelpunkt zu rücken und eine bedarfsorientierte Verkehrsplanung anzuwenden (Schwedes und Hoor 2019). Wichtig ist aber, sie nicht isoliert zu betrachten, sondern im Kontext aktueller politischer Felder wie die Transformation hin zu einer ökologisch-verträglichen sowie sozial-gerechten Zukunft in Einklang zu bringen, d. h., einen integrierten Ansatz zu verfolgen. Dies bedarf der Einbindung aller Stakeholder, um eine gemeinsame Basis für die Ansprüche an eine öffentliche Mobilität auszuhandeln. Die Mobilitätsberichterstattung kann eine solche diskursive und integrierte Entwicklung unterstützen, indem sie dazu beiträgt, die Verkehrspolitik strategisch neu auszurichten und eine nachhaltige Verkehrsentwicklung kompetent zu gestalten.

Eine strategische Ausrichtung impliziert einen integrierten, interdisziplinären Ansatz in der Planung (siehe den Beitrag von Schwedes in diesem Band). Dadurch sind die Konsolidierung und die Abstimmung der Ziele in allen Fachbereichen vonnöten, um nicht weitere politische Ziele in anderen Ressorts zu konterkarieren und die lokale Planung in sich widersprüchlich zu machen. Die strategische Ausrichtung leitet sich daher aus den Bedarfen der Menschen, der Interessen verschiedener Akteure und den gesellschaftlichen Zielen ab.

Der vorliegende Artikel befasst sich mit dem Instrument der Mobilitätsberichterstattung, mit den Zielen, Akteuren sowie Verankerungsmöglichkeiten in der Planungskultur. Die in der Praxis gesammelten Erkenntnisse entstammen dem Forschungsprojekt MobilBericht^1^, in dem erstmalig die Mobilitätsberichterstattung entwickelt und in Berlin-Pankow angewandt wurde.

## Ziele der Mobilitätsberichterstattung

Die Mobilitätsberichterstattung dient der Mobilitätsplanung. Sie ist ein Verkehrsplanungsinstrument, das als Ergänzung zur herkömmlichen Verkehrs- und Infrastrukturplanung zu sehen ist und auf eine menschengerechtere Mobilitätsplanung hinwirkt. Hierdurch wird das System Verkehr integriert gestaltet. Das derzeitige Defizit der Verkehrsplanungsinstrumente besteht in der Fokussierung auf quantitative Daten des Verkehrs, also auf die zeitliche Ausprägung von Ortsveränderungen, die den Menschen und seine Motive für die Erzeugung des Verkehrs vernachlässigt (Schwedes et al. 2018, S. 17). Durch reines „Zählen“ von Verkehr wird nicht ersichtlich, warum Menschen verkehrsrelevante Entscheidungen treffen – sei es in ihrer Verkehrsmittelwahl oder Streckenplanung. Um die Mobilität der Menschen gestalten zu können, bedarf es einer am Menschen und ihren Lebenswelten orientierten Planung. Die Mobilitätsbedarfe der Menschen – also die Anforderungen an den Möglichkeitsraum, welche zur Erfüllung der spezifischen Bedürfnisse eines Menschen oder einer Zielgruppe nötig sind – rücken dadurch in den Fokus der Mobilitätsplanung (Schwedes et al. 2018, S. 34 ff.).

Im Sinne der nachhaltigen Mobilität dienen die soziale Gerechtigkeit und ökologische Verträglichkeit (§ 1 Abs. 1 MobG BE) als normativ-leitende Ziele für die Berichterstattung. Soziale Nachhaltigkeit kann nur geschaffen werden, wenn die soziale Gerechtigkeit gestärkt wird (siehe den Beitrag von Daubitz in diesem Band). Als Bemessungsgrundlage für Gerechtigkeit in der Mobilität wird der Befähigungsansatz (capability approach) angewendet^2^, der anstrebt, dass alle Menschen mit und ohne Benachteiligungen zur Selbstbestimmung in der solidarischen Gemeinschaft gemäß ihren Fähigkeiten ein gutes Leben führen können (Sen 2012; Nussbaum 2010, 2012). Diese Teilhabe in der Planung soll die Verwirklichungschancen der Menschen und den sozialen Zusammenhalt erhöhen. Die Voraussetzungen zur Vermeidung sozialer Exklusion liegen in der Gestaltung der Öffentlichen Mobilität, die gleiche Zugangschancen zu Orten der Daseinsvorsorge und Teilhabe am öffentlichen Leben für alle bieten soll. Die Erreichbarkeit von Orten der Daseinsvorsorge erhält durch gesetzliche Festlegungen einen rechtlichen Anspruch (siehe den Beitrag von Schwedes und Ringwald in diesem Band), sodass die Mobilität zum meritorischen Gut in der Planung wird (Litman 2014). Das bedeutet, dass staatliches Handeln die Bereitstellung der Verkehrsangebote und Orte der Daseinsvorsorge fördern soll und somit nicht rein marktwirtschaftlichen Prinzipien von Angebot und Nachfrage überlässt, um den unterschiedlichen Mobilitätsbedarfen aller Menschen gerecht zu werden. In diesem Zusammenhang kann gleichzeitig ein Beitrag zu einer gesteigerten Lebensqualität geleistet werden.

Das Instrument der Mobilitätsberichterstattung verfolgt das Ziel, die Politik und die Verwaltung zu befähigen, die Verkehrsplanung strategisch auszurichten. Hierdurch haben einerseits diese Akteure die Möglichkeit, ihre politischen Ziele – bspw. Klimaziele, Luft- und Lärmreinhaltung sowie die Veränderung des Modal Splits – zu erreichen. Auf der anderen Seite wenden sich Akteure aus der Zivilgesellschaft insbesondere auf lokaler Ebene immer öfter gegen die dort von der öffentlichen Hand praktizierte Anpassungsplanung, um eine angemessene Berücksichtigung aller Verkehrsteilnehmenden im Straßenraum zu fordern (siehe den Beitrag von Schneidemesser in diesem Band). Die aufkommenden Bemühungen der Legislative in Berlin, diese Missstände durch klar definierte, gesetzlich festgeschriebene sowie ambitionierte Strategien und Maßnahmen künftig zu beheben und den Verkehr zu reorganisieren, müssen in der Exekutive ankommen (siehe den Beitrag von Kirchner in diesem Band; SenUVK 2020). Politik und Verwaltung benötigen Instrumente, mit denen auf Grundlage umfangreicher Mobilitätsuntersuchungen angemessene, lokalspezifische Maßnahmen entwickelt und in der Praxis kurz- und langfristig umgesetzt werden können. Die öffentliche Hand kann die Öffentliche Mobilität durch die Angebote an Qualität und Quantität der Verkehrsträger, des öffentlichen Verkehrs, der Stadtplanung und somit der Infrastrukturen zur Daseinsvorsorge aktiv zielorientiert gestalten.

**Figure 1:**
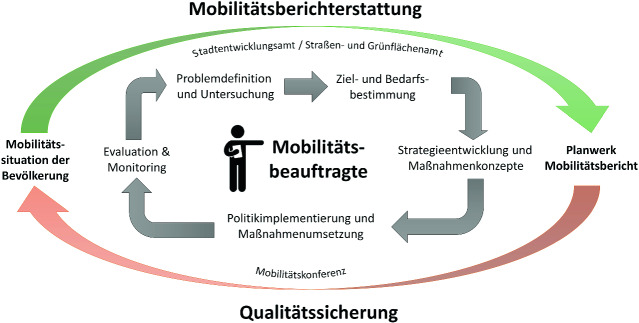

Die Mobilitätsberichterstattung ist ein fortwährender Politikzyklus (s. Abb. 1). Am Anfang stehen die Problemdefinition und die Untersuchungen, die in die Ziel- und Bedarfsbestimmung münden (Agenda Setting). Das Ziel ist es, auf der einen Seite durch Operationalisierung der Einflussfaktoren die Mobilität im Untersuchungsgebiet zu erfassen (siehe den Beitrag von Rammert in diesem Band) und sie auf der anderen Seite leitbildgerecht zu gestalten. Dies geschieht durch Aufstellung verkehrspolitischer Strategien aus dem Leitbild und der Bewertung des Status Quo mithilfe von Erhebungen und der Auswertung in einer SWOT-Analyse, deren Ergebnis wiederum ein abgeleitetes Maßnahmenkonzept ist (siehe Abschn. 2.2). Nach der Implementation in Form von umgesetzten Maßnahmen folgt die Evaluation der Mobilitätssituation, was wiederum Ausgangspunkt für einen neuen Zyklus ist. Die Umsetzung sollte in der Qualitätssicherung anhand von Evaluationskriterien bewertet werden, um in einem nachfolgenden Bericht die Zielerreichung beurteilen zu können (Schwedes et al. 2017, S. 123). Dieser Prozess wird durch eine Mobilitätskonferenz begleitet, die als beratendes Gremium die Berichterstattung unterstützt, in der Entscheidungen abgestimmt und die Vorgehensweise überwacht werden. Sie konstituiert sich aus Vertreter*innen der Verwaltung, Politik und Verbänden sowie externen Planenden und Expert*innen für eine neutrale Bewertung. In einem regelmäßigen Turnus findet die Veröffentlichung von Berichten zu den Daten, verkehrspolitischen Zielen, daraus abgeleiteten Strategien und Maßnahmen statt, deren dauerhafter Prozess eine konsequente Behandlung des Themas, die Einbindung zyklenabhängiger Schwerpunktthemen und eine fortwährende Politikevaluation gewährleistet.

Berichterstattungen für Planungszwecke zu nutzen, ist nicht neu und findet bereits im Gesundheits- und Sozialbereich Anwendung (RKI 2020; Destatis 2020). Sie sind gesetzlich festgeschrieben und dienen der Politik zur Entscheidungsfindung, um Entscheidungen kurz-, mittel- und langfristig zielgerichtet zu fällen. Die Berichterstattungen sollen auch helfen, mit neuen, für die Planung unerwarteten Ereignissen umzugehen, also auf Basis von belastbaren Daten Entscheidungen treffen zu können. Das Beispiel der Covid-19 Pandemie zeigt, dass eine unmittelbare Berichterstattung zur Erfassung der Lage vonnöten ist, um darauf aufbauend strategische Maßnahmen ableiten zu können, mit denen dem Ereignis adäquat begegnet werden kann.

## Die Einbindung von Akteuren in den Planungsprozess

**Figure 2:**
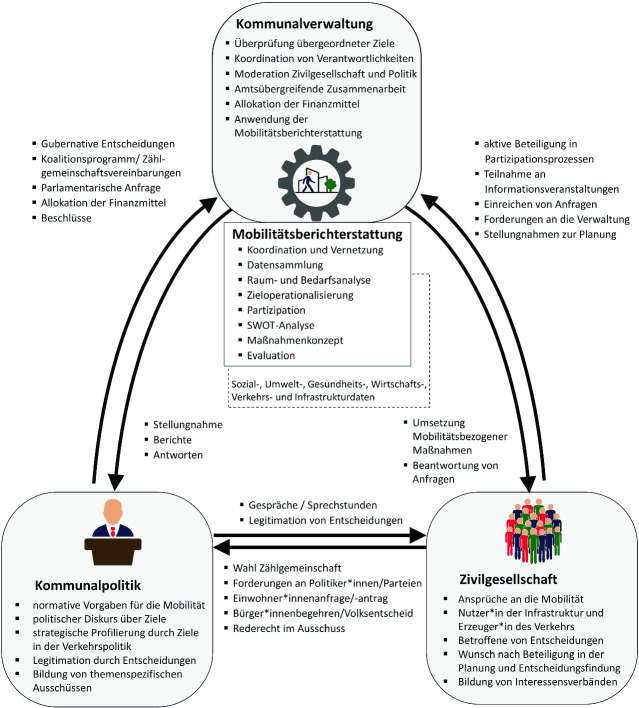

Für den partizipativen Erarbeitungs- und Planungsprozess der Mobilitätsberichterstattung müssen vielfältige Akteure involviert werden: Die Zivilgesellschaft mit ihren Bedürfnissen und daraus abgeleiteten Mobilitätsbedarfen, die Politik mit ihrer normativen Entscheidungsmacht und die Verwaltung sowie städtische Unternehmen mit der Umsetzung von politischen Entscheidungen mittels Planungen. Das Dreieck der Akteure mit ihren Aufgaben, Interessen und Kommunikationsmöglichkeiten ist in der Abb. 2 überblicksweise dargestellt.

Neben der Möglichkeit des Wahrnehmens von politischen Wahlen, dem Stellen von Einwohner*innenanfragen und -anträgen an die kommunalen Politiker*innen besteht durch die Mobilitätsberichterstattung zusätzlich die Möglichkeit, die Zivilgesellschaft aktiv in den Planungsprozess einzubeziehen. Die Distanz, die zwischen der aktuellen hoheitlichen Verkehrsplanung und den Bedarfen der Bevölkerung besteht, soll durch die aktive Mitarbeit der lokalen Bevölkerung sowie deren Interessengemeinschaften im ergebnisoffenen Erhebungs- und Umsetzungsprozess aufgehoben werden. Die Menschen und ihre unterschiedlichen Mobilitätsbedarfe – also die Teilnehmenden im Verkehr – rücken damit in den Fokus der Planung. Ihre subjektive Wahrnehmung von Mobilität wird durch Aspekte der Infrastruktur, des Verkehrs sowie den räumlichen, physischen, ökonomischen und gesellschaftlichen Rahmenbedingungen der Mobilität beeinflusst (Schwedes et al. 2018, S. 5). Sie sind die Expert*innen des Verkehrsalltags, sodass sie in die Erhebungen über den Zustand des Verkehrssystems aktiv eingebunden werden müssen (Rammert 2019b, S. 86). Durch das Empowerment des bürgerlichen Engagements zieht die Nutzer*innenperspektive stärker in die Planung ein, womit die bemängelte Dissonanz zwischen Wünschen der Bevölkerung und der Planungspraxis reduziert werden soll. Die Bewertung der Mobilität setzt sich aus der Wahrnehmung in der Bevölkerung und den Einschätzungen der Expert*innen in Politik, Verwaltung und Wissenschaft zusammen^3^, wodurch die Planenden die vielen Interdependenzen und zielgruppenspezifischen Handlungsmöglichkeiten besser nachvollziehen können. Die Transparenz im Planungsprozess und Mitwirkung der Menschen vor Ort kann zu einer höheren Legimitation der Planungsergebnisse führen, wie auch zur Akzeptanz der Maßnahmenumsetzung beitragen.

Die Bürger*innen organisieren sich zunehmend in zivilgesellschaftlichen Initiativen, wie es am Beispiel von Berlin-Pankow deutlich wird. Durch Forderungen an die Politik, Vorschläge für Umbaumaßnahmen und Öffentlichkeitsarbeit geben unterschiedliche Bürger*inneninitiativen ihre Bedürfnisse und Wünsche nach Veränderungen kund. Sie repräsentieren eine große Anzahl an Menschen mit ganz unterschiedlichen Interessen, wie z. B. Gruppen, die sich gegen Schwerlastverkehr in ihrer Wohnstraße aussprechen (Verein für nachhaltige Verkehrsentwicklung e. V. 2019), die sichere Radverkehrsanlagen fordern (Changing Cities e. V. 2020), die sich einen besseren ÖPNV im suburbanen Gebiet wünschen (Bucher Bürgerverein e. V. 2020) und Eltern, die sich für sichere Schulwege ihrer Kinder einsetzen (Kargl 2020). Jedoch beteiligen sich nicht alle Personen in organisierten Gemeinschaften, haben aber trotzdem zielgruppenspezifische Forderungen zur Anpassung des Verkehrssystems. Insbesondere sozial benachteiligte Personen haben einen geringeren Zugang zu Beteiligungsformaten (Böhnke 2010). Im Rahmen der Mobilitätsberichterstattung werden diese Personengruppen aktiv angesprochen und eingebunden, sodass sie in der Evaluation und der Umgestaltung ihres Wohnumfeldes mitwirken (Schlosberg 2007). Durch die Integration einer Vielzahl von Zielgruppen mit unterschiedlichen Anforderungen an die Mobilität werden verschiedene subjektive Mobilitätsbedarfe eines großen Bevölkerungsquerschnitts erfasst. Dies ist notwendig, um die Öffentliche Mobilität ganzheitlich und integrativ zu gestalten. Durch die Nutzung von gleichen Methoden werden die Ergebnisse vergleichbar und können kontextualisiert werden.

### Partizipation bei den Erhebungsmethoden der Mobilitätsberichterstattung

Durch partizipative Erhebungsmethoden können die subjektiven Möglichkeitsräume der Menschen in Interviews erfasst und geprüft werden. Vor allem durch die qualitativen Erhebungen können die Menschen an der Co-Produktion von Wissen zur reflektierten Bewertung des Status Quo beitragen (West et al. 2016)^4^. Gemeinsam kann der subjektive Möglichkeitsraum explorativ analysiert werden, um wichtige Einflussfaktoren zu Beschränkungen und Förderungen einer leitbildgerechten nachhaltigen Mobilität zu finden. Vor allem die qualitativen Methoden bieten einen Zugang zu Zielgruppen mit Mobilitätseinschränkungen, um potenzielle Benachteiligungen und Zugangsvoraussetzungen zu analysieren. Die Einschränkungen der Menschen können aus sozioökonomischen, räumlichen, physischen, psychischen oder sozialen Benachteiligungen resultieren. Davon sind z. B. Menschen mit (temporären) Behinderungen, Kinder, in Armut Lebende, Menschen mit Transportbedarf, Nicht-Muttersprachler*innen oder Senior*innen betroffen, denen nicht alle Verkehrsmittel und Infrastrukturen, die zeitliche Flexibilität, Orientierungsmöglichkeiten, digitalen Angebote oder Lösungen für alltägliche Konfliktsituationen im Verkehr vollumfänglich zur Verfügung stehen. Die lokalen Informationen der Anwohner*innen werden durch diese Vorgehensweise für die Planung verfügbar.

Um die Mobilitätsbedarfe in Interviews angemessen zu erfassen, wurden die Methoden der Teilnehmenden Beobachtungen und des Community Mapping angewendet (Kruse et al. 2020). Bei der Teilnehmenden Beobachtung fahren oder laufen die Personen ihre alltäglichen Wege im Umweltverbund ab, zeichnen dies per Kamera auf und werden von den Interviewer*innen begleitet. Darauf aufbauend wird im Interview die Fahrt reflektiert und neben allgemeinen Fragen zur Wahrnehmung der Mobilität ein Schwerpunkt auf individuellen Verhaltensweisen zur Bewältigung von Konfliktsituationen gelegt. In der Befragung wird auch nach einer qualitativen Bewertung des Verkehrs z. B. durch Zugänglichkeit des Verkehrsnetzes und der Verkehrsmittel sowie deren zeitliche Verfügbarkeit oder Zuverlässigkeit gefragt.

Beim Community Mapping ist die Karte das Medium, um umfangreiche Informationen zur Mobilität in den Interviews zu erhalten. Durch das Einzeichnen von Orten der Daseinsvorsorge, den Wegen dorthin un die dafür genutzten Verkehrsmittel gewinnen die Teilnehmenden ein Bild von ihrem eigenen Aktionsradius, den sie dadurch reflektieren können. Im Anschluss werden, wie bei der Teilnehmenden Beobachtung, in leitfadengeführten Interviews die Mobilität der Personen thematisiert, um möglichst viele Erkenntnisse aus den subjektiven Wahrnehmungen, verkehrsrelevanten Entscheidungen und der Verkehrspraxis zu generieren. Den Interviews können auch Anregungen für mögliche Handlungsempfehlungen entnommen werden.

Des Weiteren wurden quantitative Erhebungsmethoden eingesetzt, mit denen die bisherigen verkehrsbezogenen Erhebungen mit Aspekten der Mobilität ergänzt werden sollen. Die üblicherweise praktizierten Erreichbarkeitsanalysen sind oftmals autoverkehrsorientiert, sodass zwar für dieses Verkehrsmittel ein Einzugsbereich und schlecht erschlossene kommunale Gebiete ermittelt werden können, aber unberücksichtigt bleibt, dass das Verkehrsmittel nur einer begrenzten Zahl von Bürger*innen zur Verfügung steht. Mit der Durchführung von Erreichbarkeitsanalysen für den Umweltverbund eröffnet sich eine breitere Einsicht in die Möglichkeitsräume der Menschen, die aufzeigt, in welchen Gebieten Zugangschancen insbesondere für Menschen mit Mobilitätseinschränkungen verbessert werden müssen. Eine allgemeine Verbesserung der Erreichbarkeit im Umweltverbund wird durch die Förderung der Nahmobilität unterstützt, die durch eine bewegungsfreundliche Gestaltung des Straßenraums, einem engmaschigen Wegenetz, einer Vielfalt an möglichen Zielen und einer hohen Wohn- und Arbeitsdichte gefördert wird (Transport for London 2005; Ewing und Cervero 2010).

Mit einer Umweltgerechtigkeitsanalyse werden zudem die Einflüsse von Verkehrsfolgen in Form von Emissionen auf die Bewohner*innen untersucht. Der Staat hat die Aufgabe, die Menschen vor freiheitseinschränkenden, gesundheitsbeeinträchtigenden als auch umweltschädlichen Emissionen zu schützen und frühzeitig zu intervenieren, um negative Entwicklungen abzuwenden (Fürsorgepflicht durch Sozialstaatlichkeit, Art. 20 Abs. 1 GG). Die Emissionen des motorisierten Verkehrs haben erheblichen Einfluss auf die Gesundheit der Menschen und somit auf das subjektive Wohlbefinden (Wildavsky 1997), deshalb sind lokale Konzentrationsschwerpunkte vor allem jenseits von Grenzwerten zu identifizieren und die Belastungen zu reduzieren (Schultz 2009). Gleichzeitig wird dabei geprüft, inwieweit Menschen in sozioökonomisch prekären Lagen von den Emissionsbelastungen betroffen sind, um eine Verteilungsgerechtigkeit bei sozial-räumlichen Ungleichheiten anstreben zu können (Lippl 2003) und die Kumulation von Nachteilen zu verhindern (Preisendörfer 2002). In Armut lebende Menschen verfügen aufgrund eines eingeschränkten Möglichkeitsraums über weniger Ausweichoptionen und sind im Ergebnis besonders stark von gesundheitsschädigenden Emissionen betroffen (Schröder-Bäck 2012). Durch die Ermittlung von Emissionsquellen sollen dem Verursacherprinzip folgend insbesondere der dafür verantwortliche motorisierte Verkehr effizienter gestaltet und substituiert werden (Walker 2012).

Eine weitere Methode zur flächenhaften Messung der subjektiven Wahrnehmungen von Mobilität ist eine quantitative Befragung. Die Befragung wurde im Bezirk Pankow in Verknüpfung mit der turnusgemäßen SrV-Erhebung (System repräsentativer Verkehrsbefragungen) für Berlin durchgeführt und führt zu Erkenntnissen in den Bereichen der Wahrnehmung von Wohnumgebung, Erreichbarkeit, Mobilitätseinschränkungen und Wohlbefinden. Das Einbeziehen der SrV-Daten ermöglicht die Verknüpfung der Wahrnehmung von Mobilität mit der verkehrsrelevanten Entscheidung. Mit den Daten kann auch die bereits bestehende soziale Exklusion gemessen werden. Die Stärken und Schwächen für die Mobilität und Teilhabe der Bewohner*innen sowie deren Einflussfaktoren können auf gesamtkommunaler Ebene erfasst werden, um Problemgebiete zu identifizieren, die prioritär umgestaltet werden müssen.

**Figure 3:**
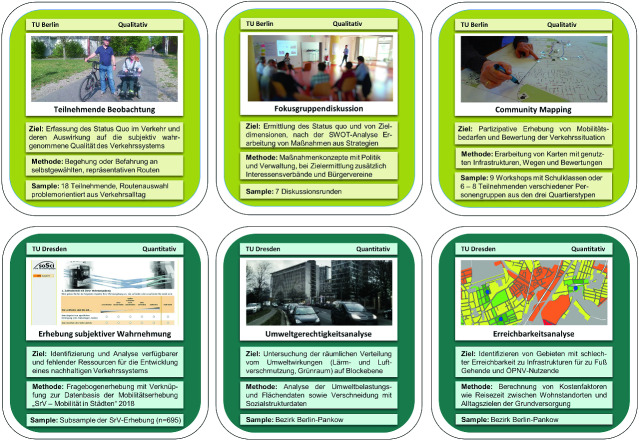

Die quantitativen und qualitativen Datenquellen sind Bestandteil eines Mixed-Methods-Ansatzes, in dem qualitative und quantitative Daten generiert, aufeinander abgestimmt und die Analyseergebnisse zusammengeführt werden (siehe Abb. 3). Ergänzt werden die Daten durch Sekundärdaten zum Verkehrssystem, die bereits erhoben und für die Mobilitätsberichterstattung von Relevanz sind. Die quantitativen Methoden ermöglichen einen flächenhaften intrakommunalen Vergleich, um Schwerpunktgebiete für Problemlagen zu Themen der Mobilität ausfindig zu machen. Damit die Wahrnehmungen und Ursachen stichprobenartig erforscht, personengruppenspezifische Bedürfnisse erfasst und weitere potentielle Einflussfaktoren auf die Mobilität identifiziert werden können, werden die qualitativen Methoden angewendet. Umgekehrt können Hypothesen, die aus den qualitativen Daten abgeleitet werden, durch quantitative Daten validiert werden, um politischen Fehlinterpretationen vorzubeugen. Die beiden Methodentypen können also in einem Themenfeld komplementär wirken und die Untersuchungsdesigns aufeinander abgestimmt sein, um die Vorteile beider methodischer Ansätze zu nutzen. Somit können nicht nur Probleme aufgedeckt, sondern auch Ursachen ergründet werden. Von der Qualität der Daten sind die Ergebnisse der Analyse und des Planungsprozesses abhängig, daher sollte für einen plausiblen und umfassenden Mobilitätsbericht ein besonderer Wert auf den Arbeitsschritt der Datenerfassung gelegt werden. Entscheidungen, die den Straßenraum betreffen, werden in verschiedenen Medien, unter der Bevölkerung und in der Politik diskutiert, sodass es wichtig ist, dass sich die Daten durch wissenschaftliche Gütekriterien wie Vollständigkeit, Nachvollziehbarkeit und Fehlerfreiheit auszeichnen (Flick 2017, S. 490).

Die Methoden sind für eine effiziente Planung der Mobilitätsberichterstattung geeignet und bieten die Möglichkeit, Schwachstellen und Chancen frühzeitig zu erkennen. Somit müssen Politik und Verwaltung schon bei den ersten Überlegungen zu den grundlegenden Anforderungen an die Berichterstattung involviert werden. Dafür eignen sich Fokusgruppen, an denen, neben Politik und Verwaltung, auch Bürger*innen und Interessenverbände teilnehmen. Es empfiehlt sich, die heterogenen Räume einer Kommune mobilitätsbezogen in homogene Räume zu separieren, um die Situation in den verschiedenartigen Raumtypen differenziert betrachten zu können. Hierdurch kann eine ganzheitliche Diskussion über Stärken, Schwächen und Visionen entstehen. In den Fokusinterviews und Gruppengesprächen wurde zudem das methodische Vorgehen des Planungsinstruments vorgestellt und diskutiert, um die Vorgehensweise zur Ermittlung der Mobilität der Bürger*innen von den Teilnehmenden überprüfen und an die örtlichen Gegebenheiten anpassen zu lassen. Die frühzeitige Beteiligung ermöglicht es, potenzielle Widerstände zu Prozessbeginn aufzudecken und im gemeinsamen Diskurs aufzuheben.

### Partizipation in der SWOT-Analyse zur Strategien- und Maßnahmenerarbeitung

Es empfiehlt sich, basierend auf den Ergebnissen der Fokusgruppen ein Grundlagenpapier zu erarbeiten, in dem die Leitlinien zur Umsetzung des Instruments und der anzustrebenden normativen Ziele beschrieben werden. Ebenso konnten für den ersten Zyklus der Mobilitätsberichterstattung (Abb. 1) Fokusthemen zur intensiven Untersuchung festgesetzt werden. Im weiteren Verlauf ist der aktuelle Stand der Berichterstattung zur Transparenz des Erarbeitungsprozesses in Ausschüssen, Verwaltungsgremien sowie über die Öffentlichkeitsarbeit im Austausch mit der Bevölkerung zu berichten und zu diskutieren. Die Abstimmung mit Politik und Verwaltung ist besonders wichtig, da die Möglichkeiten des Instruments und die damit verbundenen Chancen von allen beteiligten Akteuren verstanden und verinnerlicht werden müssen, um die Fähigkeiten durch ausreichend personelle und finanzielle Ausstattung nutzen zu können.

Nach der Datenerhebung im Mixed-Methods-Ansatz werden die Daten mithilfe der SWOT-Analyse zusammengeführt (Rammert et al. 2019; Kruse et al. 2020). Diese Analysemethode ist ein standardisiertes Verfahren zur verbal-argumentativen Bewertung der Informationen anhand eines übergeordneten Leitbilds und seinen Zielkriterien. Für die nachhaltige (urbane) Mobilität als Leitbild haben sich die Zielkriterien sozial gerecht, vielfältig und nah, umwelt- und ressourcenschonend, vielseitig erreichbar sowie gesund und sicher herauskristallisiert, mit denen strategisch-normativ das Leitbild gestaltet wird. In diesem Analyseverfahren werden nach der Bewertung des Status Quo und dem Vergleich dieses Ist-Zustands mit dem leitbildgerechten Soll-Zustand Strategien erarbeitet, um die zielorientierte Entwicklung des Verkehrssystems voranzubringen. Dafür wurden in Zusammenarbeit mit Stakeholdern aus Politik, Verwaltung und relevanten Betreiber*innen von öffentlichen Infrastruktur- und Verkehrsangeboten die Ausarbeitung von Maßnahmen vorgenommen. Im Fall von Pankow sind dies:

- Politiker*innen aus dem Bezirk
- durch die Verteilung der Verantwortlichkeiten im Verkehr Verwaltungsmitarbeitende auf Bezirks- und Landesebene
- die BVG als Nahverkehrsunternehmen und infraVelo als landeseigenes Unternehmen zur Verbesserung der Radverkehrsinfrastruktur – also städtische Unternehmen aus dem Umweltverbund

Auf Grundlage der identifizierten Strategien, werden in einem kreativen Diskurs in Form eines World Cafés konkrete Maßnahmen ausgearbeitet, priorisiert und Zuständigkeiten abgestimmt, um eine nachhaltige urbane Mobilität umzusetzen (Chang und Chen 2015).

Der politische Wille ist zum einen entscheidender Initiator zur Einführung des Instruments, darüber hinaus soll die Politik als wesentlicher Bestandteil im Erarbeitungsprozess des Mobilitätsberichts dauerhaft eingebunden werden. Grundsätzlich hat die Politik die Macht, Ziele für die Gestaltung der Öffentlichen Mobilität festzusetzen. Daher muss während der Etablierung und nachfolgend in der Umsetzung die kommunale Zählgemeinschaft beteiligt werden, damit Maßnahmen in Form von Anträgen und Beschlüssen bei politischen Versammlungen verabschiedet werden können. Die Politik erhält durch die Analyse der Untersuchungen fundierte Hinweise, auf deren beratender Grundlage Entscheidungen für leitbildgerechte Maßnahmen getroffen werden können. Die Politiker*innen müssen sich die gemeinschaftlich erarbeiteten Maßnahmen zu eigen machen und Beschlüsse verabschieden, damit die bestehenden Schwachstellen zum Erreichen des Leitbilds einer nachhaltigen urbanen Mobilität abgebaut werden. Die kommunale Verkehrspolitik kann nicht frei gestalten werden, sondern hat auch die Ziele der jeweiligen Landes-, Bundes- und EU-Verkehrspolitik zu berücksichtigen. Erst durch die Integration dieser Ziele erhält die verkehrspolitische Arbeit auf kommunaler Ebene ihre Legitimation. Die kommunale Mobilitätsberichterstattung ist die hierarchisch niedrigste, politisch-gestalterische Ebene und hat die Aufgabe, die vorgegebenen und selbstauferlegten Ziele final in der Praxis des Verkehrssystems umzusetzen.

Die kommunale Verwaltung hat als Exekutive die Aufgabe, Beschlüsse umzusetzen, zu koordinieren und zu kommunizieren. Sie ist zentraler Akteur bei der Entwicklung der Mobilitätsberichterstattung und wird damit befähigt, ihrer Aufgabe der Politikberatung nachzukommen (Brand und Michelsen 2007). Zentrales Element einer erfolgreichen Umsetzung ist eine mobilitätsbeauftragte Person oder Personengruppe, die die Aufgaben und Prozessschritte initiiert, koordiniert und alle Beteiligten aktiv involviert. Dafür sind weitere Verwaltungseinheiten als Träger öffentlicher Belange zur ämterübergreifenden Zusammenarbeit bei der Bedarfserfassung und Maßnahmenumsetzung zu beteiligen, um der integrierten Planung auf interdisziplinärer Ebene gerecht zu werden und den Umsetzungsprozess zu vereinfachen. Relevante Verwaltungseinheiten für die Öffentliche Mobilität sind u. a. die Ressorts Verkehr, Stadt, Gesundheit, Soziales und Umwelt.

Der Mobilitätsbericht soll als eines der Produkte der Mobilitätsberichterstattung von allen Akteursgruppen genutzt werden können. Um den Mobilitätsbericht allen Interessierten und Verantwortlichen auf geeignetem Weg zugänglich zu machen, sind neben einem anschaulichen und übersichtlichen Bericht auch digitale Möglichkeiten in verständlicher Sprache zu nutzen. Der Bericht soll sowohl von Politik und Verwaltung wie auch der Bevölkerung genutzt werden, um Handlungskonsequenzen für die Öffentliche Mobilität herauszustellen. Die Mobilitätsberichterstattung dient als transparente Evaluation, damit die Zivilgesellschaft den Umsetzungsprozess nachvollziehen und die Politik ihre Arbeit reflektieren und diskutieren kann. Dabei kommt die Verwaltung ihrer Aufgabe der Politikberatung nach und bezieht zugleich die Bürger*innen beim Erarbeitungsprozess mit ein.

## Einordnung der Mobilitätsberichterstattung in das Feld der Verkehrsplanungsinstrumente

Es gibt bereits mehrere Verkehrsplanungsinstrumente, mit denen auf kommunaler Ebene eine verkehrsmittelübergreifende, integrierte Planung für das Verkehrssystem durchgeführt wird (SRL 2020). Allerdings haben die in den letzten Jahrzehnten gleichbleibenden CO_2_-Emissionswerte und erzwungene Fahrverbote gezeigt, dass sie nicht effektiv angewendet werden (BMU 2019, S. 61; Europäischer Rechnungshof 2020, S. 5). Um die Verwaltung auf kommunaler Ebene zu ermächtigen, die ausgehandelten verkehrspolitischen Ziele effizient umzusetzen, müssen den Planenden geeignete Instrumente sowie eine angemessene personelle und finanzielle Ausstattung zur Umsetzung zur Verfügung gestellt werden. Die Instrumente dienen der Politik als entscheidungsunterstützende, transparente Beratung, um fundiert und zielorientiert Abwägungen zwischen Maßnahmenoptionen zur Gestaltung von Öffentlicher Mobilität treffen zu können. Der Vergleich der Mobilitätsberichterstattung mit den bisher etablierten Verkehrsplanungsinstrumenten soll aufzeigen, wodurch sich die Instrumente voneinander unterscheiden, um zu verstehen, wieso die Mobilitätsberichterstattung ein geeignetes Instrument für Kommunen zur Verkehrsplanung mit dem Fokus der Mobilität ist.

### Verkehrsentwicklungspläne und ihre Weiterentwicklung zu Sustainable Urban Mobility Plänen

In der Planungspraxis bestehen bereits mehrere Verkehrsplanungsinstrumente wie die Verkehrsentwicklungspläne (VEP), die Sustainable Urban Mobility Pläne (SUMP) und die Klimaschutzteilkonzepte Verkehr. Das älteste Instrument sind die VEP, die sich im Laufe der letzten Jahrzehnte vom klassischen Planungsinstrument weiterentwickelt haben (FGSV 2001,2013; Ahrens 2008), um die geänderten Anforderungen an die Planung und insbesondere eine nachhaltige Entwicklung sowie gesellschaftliche Wahrnehmungsveränderungen zu integrieren (Wolfram 2009). Der VEP nimmt die auf EU-Ebene entwickelten Vorgaben für den SUMP auf und setzt diese in den Planungsschritten eines Policy Cycles um (GPSM 2015; Rupprecht Consult 2019; Arndt und Drews 2019) – d. h. in einem zirkulären Planungsprozess zur Gestaltung des Verkehrs durch eine integrierte und zielorientierte Planung (Rammert 2019a). Durch diese Übernahme fand ein Assimilationsprozess der methodologischen und inhaltlichen Gestaltung von VEP hin zu SUMP statt, sodass heutzutage aufgestellte VEP immer mehr die Gestaltungsdimension der Mobilität in den Fokus rücken^5^ sowie eine ämter- und verkehrsmittelübergreifende, interdisziplinäre Betrachtungsweise anwenden. Für die Fortschreibung wird ein fünfjähriger Rhythmus empfohlen, um die Bewertung auf Grundlage der Analyse aktueller vorzunehmen (GPSM 2015), was dem zeitlichen Planungshorizont der Mobilitätsberichterstattung ähnelt. Durch das langjährige Monopol ist das Planungsinstrument etabliert und wird in verschiedenen Formen angewendet (Arndt und Drews 2019) wie z. B. beim StEP (Stadtentwicklungsplan) Verkehr in Berlin, der erstmals 2003 veröffentlicht wurde, derzeit im zweiten Fortschrittsbericht vorliegt (SenUVK 2011) und demnächst in der dritten überarbeiteten Fassung veröffentlicht wird. Die VEP bauen auf den Ergebnissen der formellen Fachpläne Luftreinhalteplan, Lärmaktionsplan und Nahverkehrsplan auf, sind aber selbst informelle Fachplanungen, die jedoch als Voraussetzung für Finanzierungen wie dem Gemeindeverkehrsfinanzierungsgesetz gelten. Sie haben eine hohe Bedeutung, da ihre Ergebnisse die verbindliche Grundlage für formelle Fachpläne wie dem Bebauungsplan und dem Flächennutzungsplan darstellen und auch als Schnittstelle zu weiteren informellen Fachplänen der integrierten Stadtentwicklungsplanung dienen (FGSV 2013).

Der VEP ist trotz alledem noch stark auf die Ziele der Kfz-Verkehrsleistungen, -fluss, -geschwindigkeit sowie -prognose und -Infrastrukturen fokussiert und bietet einen wenig integrierten Ansatz. Das übergeordnete Ziel der Nachhaltigkeit wird durch die mangelhafte Berücksichtigung der Autoverkehrsvermeidung oder umfangreichen Förderungen für den Umweltverbund wenig wirksam verfolgt. Der Zielkonflikt liegt darin, dass ein auf den Autoverkehr ausgerichtetes Verkehrssystem die Zugänglichkeit des Straßenraums für den Umweltverbund minimiert, die Nahmobilität durch Verlust von Dichte und Vielfältigkeit in der Land- und Gebäudenutzung sowie die Verkehrssicherheit senkt, die hohen Kosten und Ansprüche zur Fahrzeugführung eine Zugangsbarriere für Menschen darstellt, mehr MIV mit den Nebenfolgen höherer Emissionen entstehen lässt und demzufolge die Mobilität senkt (Litman 2011). Die VEP haben durch die Betrachtung des Verkehrs gegenüber der Mobilitätsberichterstattung den Vorteil einer besseren Skalierbarkeit, denn Verkehr lässt sich auch auf kleinerer Maßstabsebene wie Metropolregionen besser als die Mobilität einzelner Personengruppen messen, deren Bedarfe sich vor allem im Wohnumfeld ergeben. Quantitative Daten zu Verkehrsleistungen speisen den VEP, worauf aufbauend Verkehrsmodelle zur Prognose erzeugt werden. Kosten-Nutzen-Analysen für Bauprojekte sind zentraler Bestandteil der Bewertung von Projekten, was wirtschaftlich effizient ist, aber insbesondere beim zu quantifizierenden Bemessen des Nutzens kritisch zu hinterfragen bleibt. Des Weiteren werden viele Daten bezüglich des Verkehrs und der Mobilität der Menschen nur geschätzt, sodass eine Pseudoquantifizierung stattfindet, deren Aussagekraft insbesondere für eine zielorientierte Gestaltung ungewiss bleibt. Zwar wird das Instrument bereits mit „Mobility Master Planning“ zur Annäherung an die SUMP übersetzt (GPSM 2015; FIS 2018), allerdings wird die Mobilität der Menschen bis auf quantitative Befragungen nach Verkehrszwecken inkonsequent untersucht, sodass die Ursachen für den Kfz-Verkehr wenig Beachtung finden. Verkehr wird – auch durch die gerichtliche Anfechtbarkeit verkehrsplanerischer Entscheidungen – durch den Status Quo und die vermeintlich daraus ableitbaren zukünftigen Entwicklungen legitimiert (GPSM 2015, S. 21 ff.), sodass eine zielorientierte, steuernde Planung – weg von der voraussichtlichen Verkehrsentwicklung hin zu gewünschten verkehrsrelevanten Entscheidungen – nicht möglich ist. Die Verkehrsmodelle stellen die zentrale Grundlage zur Verteilung der Straßenfläche für das Kfz dar, um die restlichen Verkehrsträger zu ergänzen und daraus den Straßenraum abzuleiten (Creutzig et al. 2020, S. 2). Dem Ziel einer nachhaltigen Mobilität folgend sollten die Verkehrsmodelle aber nur ergänzend zur Dimensionierung vor allem für Infrastrukturen des Umweltverbunds genutzt werden, nachdem alle Qualitätsanforderungen z. B. zur Sicherheit und zum Komfort durch die Nutzenden berücksichtigt worden sind, um den Mobilitätsbedarfen zu entsprechen. Die Verkehrsleistungen im Umweltverbund werden jedoch mangels umfangreicher Zählungen kaum berücksichtigt. Abschließend wird die Beteiligung bei der klassischen Verkehrsplanung eher als Informationsaustausch zur Akzeptanzerhöhung der von Ingenieur*innen getroffenen Entscheidungen verstanden (Rupprecht Consult 2019, S. 10), anstatt die Anwohner*innen in der Problemanalyse als auch in der Erhebung von Daten zu involvieren und sie zur Einschätzung ihrer Mobilität zu befragen. Somit entsteht eher ein Überzeugungs- anstelle eines Aushandlungsprozesses zwischen den Verkehrsplanenden und den interessierten Bürger*innen.

Das EU-geförderte Instrument der SUMP ähnelt in Inhalt, Ziel und Aufbau stark der Mobilitätsberichterstattung (Arndt und Drews 2019). Die SUMP verfolgen mit der Erreichbarkeit und Lebensqualität sowie Nachhaltigkeit, soziale Gerechtigkeit, Gesundheitsförderung und Umweltverträglichkeit dieselben Ziele wie die Mobilitätsberichterstattung (Rupprecht Consult 2019). Der einzige, geringfügige Unterschied ist es, dass die SUMP explizit die wirtschaftliche Leistungsfähigkeit als Ziel benennen und ein ganzheitliches Finanzierungskonzept als Prozessschritt fordern, während sich die Mobilitätsberichterstattung zusätzlich dem Ziel der vielfältigen Stadt widmet und die Stadtplanung explizit einbindet. Die gesamtheitliche Betrachtung der Nachhaltigkeit beider Instrumente fördert die Interdisziplinarität und fordert die ämterübergreifende Zusammenarbeit zur Bearbeitung des Plans, wodurch eine höhere Akzeptanz und umfassendere, integrative Betrachtung geschaffen wird. Der Umweltverbund profitiert in beiden Plänen durch den Fokus auf die Umweltverträglichkeit des Verkehrs. Beide Planungsinstrumente sind in der Abgrenzung des Untersuchungsraums flexibel; doch sollten sich funktionelle und administrative Grenzen größtenteils decken, um verkehrspolitische und verwaltungshoheitliche Bezüge sowie eine Identifikation der Menschen mit dem Raum zur Bewertung ihrer lokalen Mobilität herstellen zu können. Die größte Differenz, welche die beiden Konzepte eines Verkehrsplanungsinstruments voneinander unterscheidet, ist der vorskizzierte Ablauf mit umfangreichen inhaltlichen Anforderungen des SUMP (Rupprecht Consult 2019) gegenüber dem flexiblen, problemspezifisch angepassten Ablauf und Auswahl von Erhebungsmethoden der Mobilitätsberichterstattung. An den gut strukturierten Ablauf der Verfahrensschritte zur Umsetzung der SUMP kann sich die Mobilitätsberichterstattung orientieren, aber sie sollten keine Vorschrift und auch kein Bemessungskriterium zur Qualität des Plans sein, da die Voraussetzungen in den Kommunen sehr unterschiedlich sind. Die Planungspraxis der VEP zeigt, dass ohnehin nicht alle strategisch-konzeptionellen Verfahrensschritte angewendet werden (Thiele 2018), sodass ein flexiblerer Aufbau realitätsgetreuer ist. Mit dem geringeren Aufwand ist es potenziell eine niedrigere Starthürde, einen Mobilitätsbericht aufzustellen, anstatt allen Anforderungen eines SUMP zu genügen. Zwar postulieren die SUMP, auf den Menschen fokussiert zu planen (Rupprecht Consult 2019, S. 10), doch deren Datenakquise ist kaum auf die Erfassung der Mobilitätsbedarfe ausgerichtet. Durch den Fokus auf die Verkehrserfassung anstatt von Einflussfaktoren der Mobilität werden Modellierungs- und Szenariotechniken zur Verkehrsprognose angewendet (ebd., S. 81 ff.). Die Beeinflussung der verkehrsrelevanten Entscheidungen geht damit verloren. Des Weiteren nutzt die Mobilitätsberichterstattung das beratende Gremium einer regelmäßig stattfindenden Mobilitätskonferenz mit externen Planenden und Expert*innen, um den Prozess zu begleiten, die ämterübergreifende Zusammenarbeit zu koordinieren und Umsetzungen zu evaluieren.

### Klimaschutzteilkonzepte als Verkehrsplanungsinstrumente

Die durch die Gründung der Nationalen Klimaschutzinitiative 2008 aufgekommenen und von der Bundesregierung geförderten integrierten Klimaschutzpläne haben die Möglichkeit der sektoralen Fachplanung von Klimaschutzteilkonzepten Verkehr, die auch als klimafreundliche Mobilitätskonzepte bezeichnet werden. Der Grund ihrer Einführung liegt im immer weiter steigenden Anteil des Verkehrs an den gesamten Treibhausgas (THG)-Emissionen in Deutschland durch das steigende Kfz-Verkehrswachstum und den fehlenden verkehrsplanerischen Konzepten vor allem in kleineren Kommunen (DIfU 2018). Ihr Aufbau ist nicht direkt dem Policy Cycle nach iterativ angelegt, sondern nimmt ihren Anfang beim Beschluss und endet bei der technisch-baulichen Maßnahmenumsetzung. Die klimaschutzorientierte Förderung ist zeitlich begrenzt, daher soll durch eine Verstetigungsstrategie die Kommune das Instrument selbstständig übernehmen. Die Mobilitätsberichterstattung und die Klimaschutzteilkonzepte ähneln sich im Aufbau, inhaltlich flexible Fokusthemen sowie eine dazu angepasste Erfassung und Bewertung wählen zu können. Ebenso kann bei beiden eine qualitative Ist-Analyse zur Bestandserhebung durchgeführt werden und die Ergebnisse werden im Rahmen einer SWOT-Analyse zusammengeführt. Zusätzlich zum Klimaschutzteilkonzept wird die Position der/des Klimaschutzmanagers/Klimaschutzmanagerin durch das Bundesprogramm gefördert, die sich auf den Verkehr spezialisieren kann, was mit dem Aufgabenprofil der/des Mobilitätsbeauftragten für die Mobilitätsberichterstattung korreliert (siehe Abschn. 4.1). Der Fokus der Klimaschutzteilkonzepte liegt in der Reduktion der THG (BMU 2017a; DIfU 2018). Durch den Fokus auf den Klimaschutz fällt der Mobilität der Menschen zwar eine wichtige Rolle zu, allerdings sind die Ziele der sozialen Gerechtigkeit oder Gesundheitsförderung nur Nebeneffekt und nicht zentrales Zielkriterium. Dadurch werden verkehrsrelevante Entscheidungen und die partizipative Beteiligung der Bevölkerung in der Bewertung des Status Quo weniger stark berücksichtigt.

### Bereicherung der Verkehrsplanungsinstrumente durch die Mobilitätsberichterstattung

Alle untersuchten Verkehrsplanungsinstrumente sind informell und bedürfen einen politischen Willen zur Anwendung. In den Verfahrensabläufen und der Intention, mit einer Berichterstattung einen iterativen Planungszyklus zur dauerhaften, strategischen und anpassungsfähigen Verkehrspolitik zu initiieren, gibt es viele Überschneidungen zu den anderen Instrumenten. Die turnusmäßige Fortschreibung kann bei Allen einen mittelfristigen Planungshorizont von ca. fünf Jahren aufweisen, wobei die Zyklen der VEP tendenziell langfristiger mit bis zu 15 Jahren angelegt sind; während Klimaschutzteilkonzepte, SUMP und die Mobilitätsberichterstattung auch kurzfristiger ab zwei Jahren angelegt werden können. Die Mobilitätsberichterstattung folgt nur in der SWOT-Analyse zur normativen Integration (Schwedes und Rammert 2020, S. 26 ff.) einem vorgegebenen Ablauf, wodurch mehr Flexibilität in der Wahl von Datenerhebungsmethoden und Analyseelementen besteht. Insbesondere im zweiten Teil des Politikzyklus – der Qualitätssicherung durch Maßnahmenumsetzung – können die vielen Ziele umgesetzt und der Prozess dabei unter Rücksprache aller Beteiligten auf der Mobilitätskonferenz evaluiert werden. Allerdings bestehen durch die bisher fehlende Untersuchung zur Mobilität Daten, die nun eigenständig unter erhöhtem Aufwand erhoben werden müssen. Quantitative Bewertungsmethoden wie Szenarienanalysen und Kosten-Nutzen-Analysen sind nicht vorgesehen, wodurch neoliberale Planungslogiken der ökonomischen Effizienz und Effektivität durchbrochen werden. Für die eigenständige Betrachtung des Wirtschaftsverkehrs oder die Berechnung von THG- Emissionseinsparungen müssen dadurch auf andere, bisher etablierte Verkehrsplanungsinstrumente zurückgegriffen werden. In der Betrachtung von Öffentlicher Mobilität stehen für die Mobilitätsberichterstattung daher weniger die kostenintensiven, infrastrukturschaffenden Maßnahmen, sondern mobilitätsbeeinflussenden und verkehrssteuernden Maßnahmen im Fokus, die dem geringen finanziellen Spielraum der Verwaltungen angemessen sind. Dafür ist ein verändertes Verständnis von Öffentlicher Mobilität sowie deren Ziele vor allem in politisch-administrativen Akteursgruppen vonnöten. Die Mobilitätsberichterstattung folgt dabei den Empfehlungen zur Anforderung für die Ausgestaltung der Planungsinstrumente wie z. B. (langfristige) strategische Konzepte, intersektorale Konzepte wirksamer Fachpolitiken, Konzepte des Flächen-, Klima- und Umweltschutzes oder Konzepte der Organisation und des Managements (SRL 2020, S. 61).

Eine vergleichende Bewertung des neuen Verkehrsplanungsinstruments mit den bereits etablierten erfolgt abschließend aus der Perspektive der einzelnen Akteursgruppen:

*Aus Sicht der Bevölkerung:* Durch den Ansatz weg von der Verkehrsobjektzählung und hin zu der Erfassung subjektiver Mobilitätsbedarfe bilden die Menschen wieder den Untersuchungsschwerpunkt der Verkehrsplanung. Zwar zielen alle Planungsinstrumente auf die Mobilitätssuffizienz ab, aber kein Instrument betrachtet dafür fokussiert die Mobilität der Menschen. Die partizipative Einbindung der Bevölkerung und Interessengruppen bzw. -verbände in der Problem- und Zieldefinition, Status-Quo-Erhebung und Maßnahmenentwicklung ermöglicht der Planung eine höhere demokratische Akzeptanz und somit ein geringeres Konfliktpotenzial zu erlangen. Insbesondere das Einbringen der Nutzer*innenperspektive von mobilitätseingeschränkten Personen in die Bewertung des Umweltverbunds und der Nahmobilität kann einen Beitrag zum sozialen Zusammenhalt leisten und soll die Nutzung dieser Verkehrsmittel fördern. Die Mobilitätsberichterstattung hebt die sozialen und ökologischen Ziele wieder in den Vordergrund, um sich von der ökonomisch-dominierten Denkweise lösen zu können und den gesellschaftlichen Diskurs der Art und Weise des Zusammenlebens wieder zu stärken.

*Aus Sicht der Verwaltung:* Der Verwaltung wird ermöglicht, mit der Mobilitätsberichterstattung ihre Aufgabe der Politikberatung und zielorientierten Verkehrsplanung umfangreich wahrzunehmen. Die ämter- und abteilungsübergreifende Zusammenarbeit bildet eine Chance der fachlichen Integration, die auftretenden Fragestellungen interdisziplinär angehen zu können und gemeinsam zu kreativen und geeigneten Lösungen zu kommen (Schwedes und Rammert 2020, S. 34 ff.). Allerdings sollten diese neuen Möglichkeiten und Kooperationen als Gewinn und nicht als zusätzliche Belastung verstanden werden, denn ansonsten sind Widerstände zu erwarten, die den Prozess der Zusammenarbeit weiter erschweren (Alt 2018, S. 10 ff.). Durch die Einstellung einer Person für das Mobilitätsmanagement kann die Vernetzung zentral organisiert und eine Ansprechperson für die Bevölkerung im Amt geschaffen werden, um andere Abteilungen zu entlasten. Die frühzeitige Einbindung von kommunalen Unternehmen in verkehrspolitische und -planerische Prozesse ermöglicht eine konfliktpräventive Betrachtung der Maßnahmenumsetzung, die in der Vergangenheit oftmals dadurch entstand, dass der Exekutive Vorgaben zur Umsetzung vorgesetzt wurden.

*Aus Sicht der Politik:* Die Politik erhält durch den Mobilitätsbericht eine umfassende Grundlage, auf der Abwägungen für verkehrspolitische Entscheidungen getroffen werden können. Dadurch, dass die Mobilität der Menschen wieder in den Fokus verkehrspolitischer Entscheidungen rückt, wird aus der Mobilitätspolitik unter der Berücksichtigung sozialer Gerechtigkeit eine Teilhabepolitik, die einen breiten gesellschaftlichen Diskurs ermöglicht. Die Beteiligung von Personengruppen mit Mobilitätseinschränkungen schafft eine inklusive Politik, welche die wahrgenommene Distanz zwischen Bevölkerung und Politik verringern soll und die Demokratie stärkt (Böhnke 2010). Durch die politisch und räumlich integrierte Verkehrsplanung kommen alle beteiligten Akteurs- und Interessengruppen der involvierten Planungsebenen zusammen, sodass politische Entscheidungen von einer größeren Gruppe und auf verschiedenen Ebenen politisch-administrativer Einheiten getragen werden (Schwedes und Rammert 2020, S. 30 ff.).

Zur Wahrnehmung und Umsetzung der Gestaltungsmöglichkeiten Öffentlicher Mobilität durch die Verwaltung und Politik bietet sich die Mobilitätsberichterstattung durch ihre Ziele, der Analyse von Mobilitätsbedarfen und eine gute Übersicht über die Handlungsfelder als geeignetes Instrument zur Politikformulierung und -implementierung an. Sie kompensiert die derzeit bestehenden Defizite der Planungsinstrumente, den Verkehr anstatt die Mobilität als Gestaltungsdimension im Verkehrssystem zu betrachten. Dies fordert einen entsprechenden verkehrspolitischen und -planerischen Gestaltungswillen. Die nicht konsequente Umsetzung von verkehrspolitischen Zielen in Strategien und deren Maßnahmen soll durch den Einsatz von Pull- und Push-Maßnahmen behoben werden, wodurch die Lücke an Handlungsmöglichkeiten geschlossen wird.

## Strukturelle Verankerung einer Mobilitätsberichterstattung

Eine zentrale Herausforderung zur Etablierung eines neuen Verkehrsplanungsinstruments ist die Verankerung in die bestehende Planungspraxis und die Erhöhung der Akzeptanz für neue Impulse. Zunächst müssen die Zieladressaten aus Politik und Verwaltung davon überzeugt werden, dass Handlungsbedarf besteht, um die Verkehrspolitik strategisch neu auszurichten. Zudem muss die Planungspraxis den Umgang mit einem neuen Planungsinstrument erlernen, um es optimal anwenden zu können. Dies setzt voraus, das Mobilität von Verkehr thematisch abgegrenzt und ein integrierter Ansatz zur Bewältigung des Problems angesetzt wird. Es gilt die Öffentliche Mobilität zu gestalten. Die Berichterstattung leistet für die Verkehrsplanung die Erhebung wichtiger und aktueller qualitativer und quantitativer Daten und Informationen zum Zustand des Verkehrssystems, überprüft und überarbeitet das Leitbild und deren Zielkriterien in Zusammenarbeit mit allen Akteuren und bringt abschließend Soll- und Ist-Zustand durch die Operationalisierung in Strategien und Maßnahmen zusammen. Die Verwaltung ist gefragt, die Ressourcenallokation zur Umsetzung geeigneter Maßnahmen zur Anpassung des Verkehrssystems an die leitbildgerechten Mobilitätsbedarfe der Bürger*innen vorzunehmen.

### Personelle und finanzielle Verankerung der Mobilitätsberichterstattung

Den größten Bedarf zur erfolgreichen Umsetzung besteht in der personellen und finanziellen Ausstattung des Instruments als auch der daraus resultierenden Maßnahmen für das verkehrspolitische Handeln. Die Akzeptanz zur Gestaltung einer nachhaltigen Mobilität besteht bereits größtenteils, doch werden gleichzeitig immer wieder die Hindernisse deutlich, die den Prozess verlangsamen und die Wirkung entkräften (FES 2019). Eine Verpflichtung von allen Akteuren sowie finanzielle und personelle Zusagen aus der Politik zur Institutionalisierung dieser Planung ist notwendig, um die erfolgreiche Umsetzung auf allen Ebenen zu gewährleisten (Europäischer Rechnungshof 2020, S. 5). Finanziell sollten neben den eigenen Ressourcen auch Fördermittel akquiriert werden. Neben maßnahmengebundenen Förderungen kann auch personell beispielsweise aus der Nationalen Klimaschutzinitiative ein*e Klimaschutzmanager*in mit Hauptaufgabengebiet Mobilität eingeworben werden (BMU 2017b). Weitere nationale oder länderspezifische Fördermöglichkeiten zur Aufstellung und Betreibung der Mobilitätsberichterstattung sowie zur Umsetzung von Maßnahmen fördern die Umsetzung des Instruments und zeigen den politischen Willen zur Gestaltung der Mobilität.

Zur erfolgreichen Verankerung des Planungsinstruments in der Verwaltung bedarf es einer Person, die das neue Planungsinstrument beherrscht. Sie hat die Aufgabe, weitere Ansprechpartner*innen, Initiator*innen und Verantwortliche für die Belange der Mobilität in der Planung zu gewinnen und die Umsetzung des Instruments und seiner Maßnahmen zu koordinieren. Die Person verfolgt das Ziel, die im Mobilitätsbericht festgeschriebenen Maßnahmen umzusetzen, um hierdurch der Öffentliche Mobilität Gestalt zu geben. Hierfür gilt es, Kommunikationskanäle aufzubauen, um Verantwortlichkeiten, Budgets, Finanzierungsquellen und etwaige andere Hemmnisse zu thematisieren und zu lösen. Das neue Handlungsfeld der Mobilität sollte nicht in die bestehenden Amtshierarchien subordinativ eingeordnet werden, um dem Mobilitätsbeauftragten die interdisziplinären als auch ämterübergreifenden Handlungsmöglichkeiten und Kompetenzen zu geben, die benötigt werden, um eine kooperative Zusammenarbeit zu initiieren. Dementsprechend sollte der Bereich als Stabsstelle der Amtsleitung mit engen Beziehungen zur bisherigen Verkehrsplanungsabteilung eingerichtet werden. Die/Der Mobilitätsbeauftragte sollte in allen kommunalen Austauschformaten mit einem Bezug zur Mobilität eingebunden werden, um auf der einen Seite selbst einen Überblick über den Status Quo der Entwicklungen gewinnen und auf der anderen Seite Maßnahmen einbringen und Projektpartner*innen einbeziehen zu können.

### Rechtliche Verankerung der Mobilitätsberichterstattung

Idealerweise ist die Mobilitätsberichterstattung rechtlich verpflichtend verankert, damit sie mit den notwendigen finanziellen Mitteln zur Umsetzung ausgestattet wird. Dadurch wird das Unterlassen der Einleitung notwendiger Schritte zur Umsetzung der Planungen rechtlich anfechtbar. Der öffentlichen Hand kommt eine wichtige Aufgabe in der Gestaltung der Mobilität zu, die in Form der Daseinsvorsorge rechtlich bereits impliziert festgeschrieben wurde. Wenn das Instrument einklagbar ist, wird eine flächendeckende Anwendung möglich. Als Vorbild kann die Gesundheitsberichterstattung dienen, die in den föderalen Gesundheitsdienst-Gesetzen aller Bundesländer rechtlich verankert ist. Darin sind die inhaltlichen Anforderungen an den Umfang und die Prozessschritte der Berichterstattung festgesetzt. Die Grundlage dafür wurde aber bereits vor über 20 Jahren gelegt (SVRKAiG 2000).

In Berlin beispielsweise bildet das neue Mobilitätsgesetz (MobG BE) eine rechtliche Grundlage, welche die Verankerung der Mobilitätsberichterstattung ermöglicht. Im Mobilitätsgesetz wurde erkannt, dass das System Verkehr auf Grundlage der Mobilitätsbedarfe der Menschen gestaltet werden soll (§ 4 Abs. 1 MobG BE) – auch wenn ein Mobilitätsmanagement nicht Einzug in das Gesetzwerk gefunden hat. In § 1 Abs. 3 MobG BE wird bereits explizit erwähnt, dass innovative Mobilitätskonzepte erprobt und genutzt werden sollen, woraus die Durchführung der Mobilitätsberichterstattung hervorgehen kann. Eine explizite rechtliche Legitimation zur Anwendung des neuen Verkehrsplanungsinstruments ist § 16 Abs. 5 MobG BE, in dem Folgendes gesetzlich festgehalten ist: „Alle zwei Jahre ist ein Fortschrittsbericht zur Umsetzung vorrangiger Maßnahmen zu erarbeiten. Mit Vorliegen quantitativer Erkenntnisse aus regelmäßig durchzuführenden Befragungen der Wohnbevölkerung und Zählungen ist ein Evaluationsbericht zu fertigen, der über die Erreichung der mit dem StEP Mobilität und Verkehr beschlossenen Qualitäts- und Handlungsziele und die Umsetzung der in ihm enthaltenen Maßnahmen berichtet. […] Die Fortschreibung hat spätestens nach zehn Jahren zu erfolgen.“ Die Aussage ist auf den auf Senatsebene erarbeiteten Berliner VEP – der StEP Mobilität und Verkehr – bezogen. Vor allem der neue gesetzlich festgelegte Fokus auf Mobilität kann durch die Mobilitätsberichterstattung gefördert werden. In Zukunft würde eine explizite Festsetzung der Planaufstellungspflicht zur Etablierung des neuen Planungsinstruments und der Umsetzung der in § 1 Abs. 1 MobG BE genannten Ziele beitragen. Zwar ist die Verkehrsplanung auf Bezirksebene in Berlin noch nicht verpflichtend, doch bietet sich die Möglichkeit, in Anknüpfung an §§ 4 Abs. 1 und 16 Abs. 5 MobG BE das Instrument zu legitimieren und durch Mittel des Senats zu unterstützen. Insgesamt wird im Mobilitätsgesetz mehrmals auf die Evaluierung verschiedener Einflussfaktoren der Mobilität hingewiesen:

- Verkehrssicherheit: §§ 17 Abs. 4, 21 Abs. 5 und zum Radverkehr 37 Abs. 5 MobG BE
- Zustand der Verkehrsträger des Umweltverbunds: §§ 22 Abs. 4 & 5 MobG BE
- Nahverkehr: § 28 Abs. 11 MobG BE
- Schienenverkehrsinfrastruktur: § 31 Abs. 2 MobG BE
- Radverkehr: §§ 39 Abs. 5 & 6 MobG BE
- Fußverkehr: § 52 Abs. 3 MobG BE

All diese Evaluierungserfordernisse bieten die Möglichkeit, die Mobilitätsberichterstattung als Verkehrsplanungsinstrument zur Erfüllung der gesetzlich festgeschriebenen Aufgabe einzusetzen.

### Planungspolitische Verankerung der Mobilitätsberichterstattung

Des Weiteren ist bei der Institutionalisierung darauf zu achten, dass unter Berücksichtigung einer vollwertigen Berichterstattung die Fortschreibung gewährleistet wird. Erst unter dieser Bedingung ist eine Evaluation möglich, wodurch eine transparente Bewertung der bisherigen Maßnahmen stattfinden kann. Durch das Fortschreiben wird aus einem Bericht eine dauerhafte Berichterstattung, die es ermöglicht, das Agenda Setting flexibel auf akute verkehrspolitische Themen auszurichten und Erhebungsmethoden dahin gehend anzupassen. Die Anpassungen verkehrspolitischer Ziele durch neue Entwicklungen lassen sich im nächsten Planungszyklus durch wieder durchzuführende Diskussionen darüber verständlich begründen und ermöglichen einen offenen Umgang des demokratischen Konsensfindungsprozesses. Erst durch die turnusgemäße Überprüfung und Überarbeitung wird ein Prozess ermöglicht, der die Planungsdynamiken ausreichend berücksichtigt und eine kontinuierliche, zielorientierte Umsetzung verkehrspolitischer Ziele ermöglicht. Voraussetzung dafür ist aber eine vollständige Institutionalisierung u. a. durch die Fortführung von Personalstellen, die ausreichend finanzielle Ausstattung sowie die Ausstattung mit den notwendigen Kompetenzen.

Die Mobilitätsberichterstattung wird bei der Planaufstellung und Umsetzung von Maßnahmen durch eine turnusmäßige Mobilitätskonferenz begleitet, die zur Beratung und Entscheidungsunterstützung im Prozess agiert. Die im Mobilitätsbericht erarbeiteten Maßnahmenkonzepte sollen in der Praxis umgesetzt werden, daher ist eine Aufgabe der Mobilitätskonferenz, die Handlungsempfehlungen zu bewerten und mit den Verantwortlichen über die Umsetzung zu diskutieren. Gleichzeitig dient sie als ämterübergreifende Informationsveranstaltung über den aktuellen Fortschritt im Politikzyklus. Neben den Amtsvertreter*innen und Politiker*innen sind weitere Vertreter*innen anderer Kommunen eingeladen, die sowohl mit ihrer Expertise den Prozess und die Diskussionen weiterentwickeln sowie als Multiplikator*innen in weiteren Kommunen wirken können.

#### Fazit

Die Mobilitätsberichterstattung ermöglicht es, die Gestaltungsdimension der Mobilität erstmalig vollständig in den verkehrsplanerischen Fokus zu rücken. Das neue Verkehrsplanungsinstrument berücksichtigt alle Rahmenbedingungen der Mobilität. Die Gestaltungsdimension des Verkehrs wird nur als Folge der aus den individuellen Mobilitätsbedarfen heraus getroffenen verkehrsrelevanten Entscheidungen betrachtet und stellt damit nicht mehr den zu gestaltenden Untersuchungsschwerpunkt dar. Sie bricht mit der Maxime, die Planungen an aktuelle Verkehrsentwicklungen im Modal Split anzupassen. Diese Vorgehensweise unterscheidet das Instrument von den bisher angewandten Planungsinstrumenten wie den VEP, den SUMP oder den Klimaschutzteilkonzepten Verkehr. Die Verkehrsplanung wird damit befähigt, das übergeordnete Leitbild einer nachhaltigen Verkehrsentwicklung nach allen Zielkriterien – neben den ökonomischen also insbesondere den sozialen und ökologischen – strategisch-zielorientiert umzusetzen und öffentliche Mobilität dementsprechend zu gestalten. Durch die partizipative Einbindung der Bevölkerung in die Planungsschritte und die Bewertung des Verkehrssystems aus der Nutzer*innenperspektive sowie der Zusammenarbeit zwischen Verwaltung und Politik wird die Mobilität in einer Multi-Akteurs-Partnerschaft interdisziplinär gestaltet. Damit können bestehenden Legitimations- und Kooperationsproblemen in der Verkehrsplanung wie z. B. Proteste der Bevölkerung oder Umsetzungsschwierigkeiten von Maßnahmen entgegengewirkt werden kann. Die Nutzung mobilitätsfokussierter Erhebungsmethoden zeigt neue Perspektiven auf, wie das Verkehrssystem von verschiedenen Personengruppen – mit und ohne Mobilitätseinschränkungen – wahrgenommen wird. Die Mobilitätsberichterstattung verfolgt eine am Menschen orientierte Verkehrsplanung, die den Befähigungsansatz dazu nutzt, gerechtere Verhältnisse zwischen den Verkehrsteilnehmenden zu schaffen und allen die Möglichkeit zu geben, am Verkehr teilzuhaben. Die Planenden rücken somit von ihrer Rolle ab, Vorgaben zu machen, wie das Verkehrssystem am besten funktioniert, sondern beziehen die Bürger*innen als Verkehrsexpert*innen des Alltags ein, das System gemeinsam mit allen Stakeholdern des Verkehrsplanungsprozesses zu gestalten.

Aber um diesen Paradigmenwechsel zu erreichen, bedarf es eines politischen Willens und der Offenheit der Verwaltung. Zwar ist das politische Klima der Verkehrswende derzeit für Veränderungsprozesse offen, aber die Verwaltung zeigt sich in ihren Vorschriften und Regularien wenig experimentierfreudig. Eingefahrene Prozesse und Strukturen müssen kritisch reflektiert werden, um die Verkehrsentwicklung in Richtung der Ziele auf Bundes-, Landes- und Kommunalebene zu lenken. Ein neues Verkehrsplanungsinstrument ist weniger eine Belastungsprobe für den dynamischen, menschenverursachten Verkehr, sondern für die starren Prozesse der administrativen Exekutive. Als Herausforderung zur Initiierung eines neuen Instruments gilt die institutionelle Verankerung in Form von Personal und Haushaltsmittel, die dafür zur Verfügung gestellt werden müssen. Durch die ämter- und abteilungsübergreifende Zusammenarbeit müssen neue Formate erprobt werden, um die Mobilität interdisziplinär gestalten zu können. Vonseiten der Legislative gibt es durch das neu aufkommende Mobilitätsgesetz neue Anknüpfungspunkte, den Planungsprozess für neue Instrumente zu öffnen. Die Form der Berichterstattung im Rahmen eines Politikzyklus ist für eine dauerhafte, iterative, zielorientierte, evaluierbare, Integrierte Verkehrsplanung geeignet. Die Verwaltung kann vom partizipativen Ansatz profitieren, indem die Interessen von Bürger*innen – für deren Wohl geplant wird – und Politiker*innen – die sich für das Gemeinwohl einsetzen – gemeinsam transparent diskutiert werden und Entscheidungen für Veränderungen abgewogen werden.

Die Mobilitätsberichterstattung erfasst erstmalig vollumfänglich die Mobilität im System Verkehr mit für die Verkehrsplanung neuen Erhebungsmethoden und erweitert die Gestaltungsperspektive der Verwaltung, die Öffentliche Mobilität leitbildgerecht zu entwickeln. Obwohl die Mobilität den subjektiven Möglichkeitsraum der Menschen beschreibt, sind die öffentlichen Institutionen zentrale Akteure ihrer Ausprägung, da die Rahmenbedingungen von ihnen geschaffen und beeinflusst werden. Angesichts dieser Einflussmöglichkeiten ist es wichtig, alle Stakeholder einzubinden und gemeinsam die Gestaltung der Öffentlichen Mobilität auszuhandeln.

## Footnotes

1 Gefördert durch die Initiative Zukunftsstadt vom Bundesministerium für Bildung und Forschung (BMBF) als Verbundvorhaben (TU Berlin, TU Dresden, Stadtentwicklungssamt Pankow).

2 Eine Übersicht über weitere Gerechtigkeitsansätze und deren Operationalisierung für die Verkehrsplanung bieten Creutzig et al. (2020).

3 Diese Konstellation wird als Multi-Akteurs-Partnerschaft bezeichnet (GIZ o. J.).

4 Für eine ausführliche Beschreibung der qualitativen Erhebungsmethoden und der anschließenden SWOT-Analyse siehe Kruse et al. (2020).

5 Siehe die Umbenennung des StEP Verkehr Berlin zu StEP Mobilität und Verkehr mit der dritten Fassung, § 16 MobG BE.
